# Diagnosis of an Occult Aortoenteric Fistula and Treatment of a Silent Threat

**DOI:** 10.7759/cureus.80937

**Published:** 2025-03-21

**Authors:** Abdelkader Abderrahmane, Hamza Retal, Mieke Cannie, Mohamed Khalil Khabet, Redouane Kadi

**Affiliations:** 1 Radiology Department, Brugmann University Hospital, Brussels, BEL; 2 Radiology Department, Helora University Hospital, Nivelles, BEL; 3 Radiology Department, Erasmus University Hospital, Brussels, BEL

**Keywords:** aortic aneurysm, aortic endoprosthesis, aortoenteric fistula, ct imaging, gastrointestinal hemorrhage

## Abstract

An aortoenteric fistula (AEF) is an abnormal communication between the aorta and the gastrointestinal tract, which can lead to severe gastrointestinal bleeding, sepsis, and high mortality if not promptly diagnosed and surgically managed. Its occurrence, particularly in patients with prior aortic surgery, presents considerable diagnostic and therapeutic challenges.

This case report describes an iatrogenic aorto-duodenal fistula following surgical intervention at the aortoiliac bifurcation, highlighting the complexity and rarity of this entity. The clinical presentation of AEF is often insidious, delaying diagnosis. While no imaging modality provides definitive confirmation, computed tomography (CT) remains the most effective and widely utilized tool for detection. Key imaging findings in patients presenting with gastrointestinal bleeding include contrast extravasation from the aorta into the intestinal lumen, as well as the presence of enteric material or gas within the periprosthetic space. Early recognition of these radiologic indicators is critical to timely intervention. Surgical repair remains the mainstay of treatment, with the primary objectives being hemostasis, aortic reconstruction, and infection control. The choice of surgical approach is dictated by the location and extent of the fistula, as well as the patient’s overall clinical status. Despite advancements in imaging and surgical techniques, AEF continues to pose a major diagnostic and therapeutic challenge. A high index of suspicion and familiarity with its clinical and radiologic presentation are essential for early recognition and optimal management.

## Introduction

An aortoenteric fistula (AEF) is a life-threatening condition associated with a near-100% mortality rate if left untreated, necessitating urgent surgical intervention. An AEF is classified as either primary or secondary.

Primary AEFs are exceedingly rare and are predominantly linked to pre-existing aortic aneurysms. Approximately 250 cases have been documented, with an estimated annual incidence of 0.007 per million [1].

Conversely, secondary AEFs are more prevalent and typically arise as complications following aortic reconstruction, with or without an endovascular prosthesis. Their incidence ranges between 0.6% and 2% annually, with an increased risk in patients who have undergone open aortic surgery compared to those treated with endovascular techniques [1].

When evaluating AEF, it is imperative to consider periprosthetic infection without fistulization as a key differential diagnosis. Additionally, conditions such as retroperitoneal fibrosis, mycotic aneurysm infection, aortitis, and peri-aortitis must be carefully assessed, as their management strategies differ substantially from those of AEF [2].

The management of AEF depends largely on the timing of diagnosis and the patient's clinical stability. Early diagnosis and intervention are essential to improving survival rates. Treatment typically involves surgical resection of the fistula and infected tissue, followed by aortic repair. In some cases, endovascular stent grafting may be an option, especially for patients who are not candidates for open surgery. However, surgical repair remains the gold standard, with the goal of controlling hemorrhage, managing infection, and restoring aortic integrity. Adjunctive antibiotic therapy is often necessary, particularly in the case of infected grafts or mycotic aneurysms. Despite the advances in surgical techniques, the overall prognosis remains poor, especially in patients with delayed presentation or those with extensive comorbidities [3].

## Case presentation

An 87-year-old patient with a significant cardiovascular and pulmonary history, including hypertension, decompensated heart failure, previous abdominal aortic aneurysm repair with an aortobifemoral prosthesis 10 years prior, chronic obstructive pulmonary disease (COPD), hypothyroidism, dementia, and a femoral neck fracture treated with a prosthesis complicated by infection and hematoma one year prior. The patient presented to the emergency with bronchopneumonia and progressive clinical deterioration.

On admission, the patient presented with worsening dyspnea, productive cough, and general fatigue. Clinical examination revealed a focal pulmonary abnormality in the left lower lung field. Abdominal evaluation showed a soft, depressible, and painless abdomen, with no history of melena or gastrointestinal bleeding. Neurologically, the patient exhibited left hemiparesis, likely secondary to a previous cerebrovascular event. Laboratory findings showed a mild inflammatory response, with leukocytosis (14,000/mm³), elevated C-reactive protein (CRP) at 85 mg/L, and normocytic normochromic anemia (hemoglobin 10.2 g/dL).

Chest radiography revealed pulmonary consolidation and an associated pleural effusion, predominantly in the left lower lung field, consistent with bronchopneumonia. Additionally, mediastinal widening was noted, raising concern for an underlying vascular abnormality. Given the patient’s history of aortic aneurysm repair and the presence of mediastinal widening, a thoracoabdominal CT angiography was performed to assess for potential aortic pathology.

CT imaging confirmed the consolidation and pleural effusion while demonstrating extensive atherosclerotic remodeling of the thoracic aorta and coronary arteries. A fusiform aneurysm of the descending thoracic aorta at the T7-T8 level measured 50 mm in the anteroposterior diameter while the ascending aorta measured 40 mm. At the abdominal level, an aneurysmal extension measuring 49 mm was identified below the renal arteries, with an aortobifemoral prosthesis in place.

**Figure 1:**
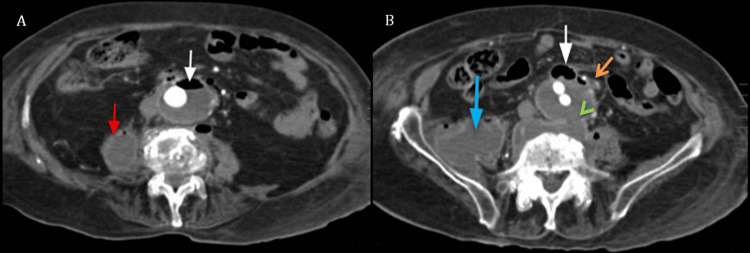
Axial slices of an abdominal CT angiography Image A. Demonstrates an aortic aneurysm with an air-fluid level on its anterior aspect (white arrow) and a collection within the right psoas muscle (red arrow). Image B. Highlights the same anterior air bubble (white arrow) in close proximity to a small intestinal loop at the duodenojejunal junction, adjacent to the aneurysm (orange arrow). The lateral collection extends along the trajectory of the psoas muscle (blue arrow). Additionally, an anterior paravertebral collection is noted, associated with a focal defect in the aortic wall directly in front of it (green arrowhead).

Of particular concern, a focal air-fluid level was detected on the anterior aspect of the abdominal aorta, adjacent to intestinal loops at the duodenojejunal junction, raising suspicion for a subtle aortoenteric communication (Figure 1).

**Figure 2:**
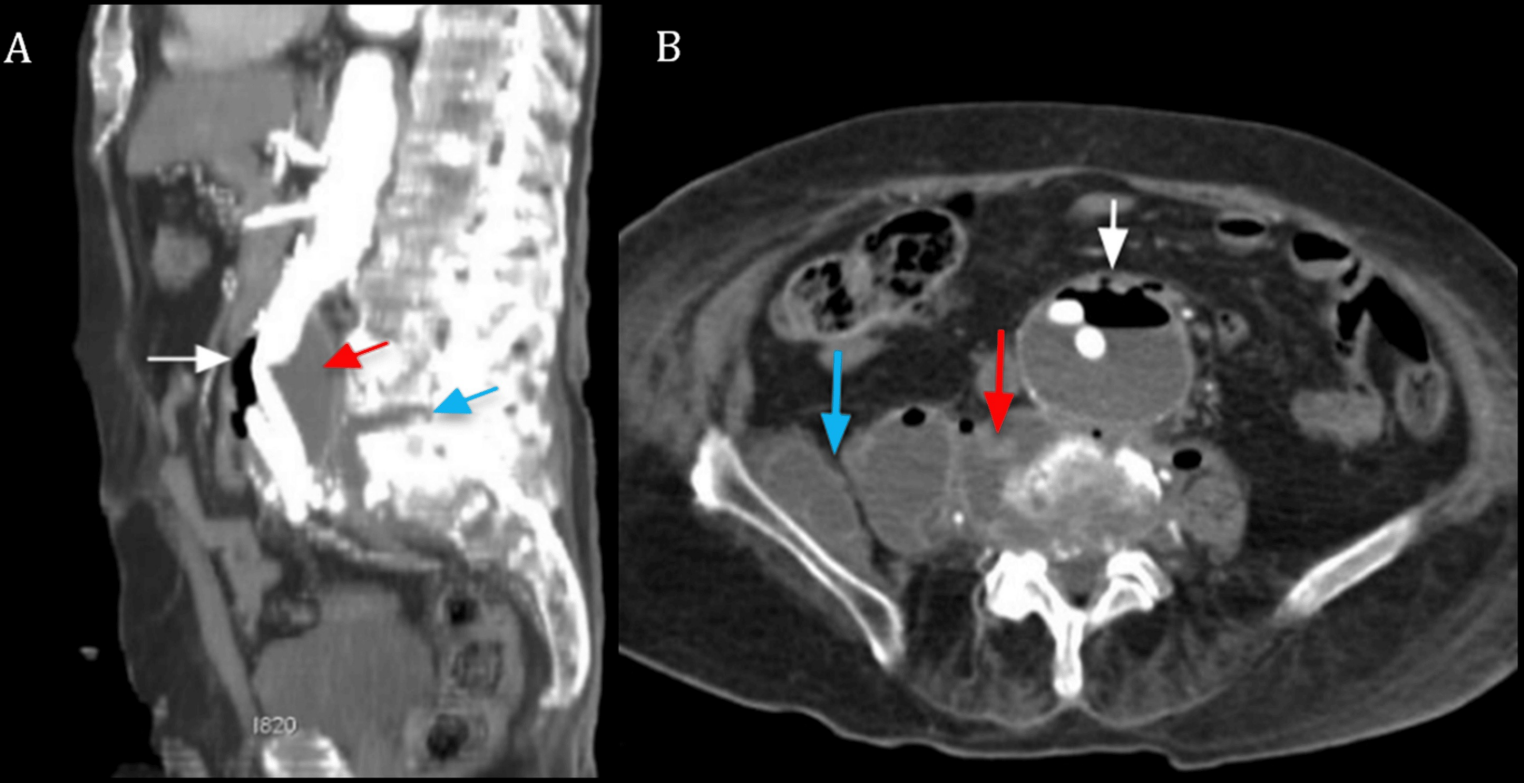
Abdominal CT angiography Image A. MIP sagittal CT view depicts an abdominal aortic aneurysm located below the renal arteries, with an air-fluid level (white arrow). Posteriorly, a paravertebral collection (red arrow) is identified, associated with L5-S1 disc destruction, and a collection (blue arrow), indicative of spondylodiscitis, in close proximity to the aneurysm. Image B. Axial CT slice demonstrates an anterior communication between the aneurysm’s anterior wall and an adjacent small intestinal loop (white arrow). Posteriorly, the paravertebral collection (red arrow) extends into the surrounding soft tissues, involving the right psoas and iliacus muscles (blue arrow).

Additionally, a periaortic collection near the iliac bifurcation extended into both iliac fossae, more pronounced on the right side, with multiple air bubbles demonstrating contrast enhancement, highly suggestive of an infectious process. Further findings included a collection near the right hip joint extending into the right psoas muscle, as well as spondylodiscitis at L5-S1, likely related to the previously infected hip prosthesis (Figure 2).

Following a multidisciplinary discussion involving vascular surgeons, interventional radiologists, and intensivists, the decision was made to adopt a conservative management approach due to the patient's advanced age, frailty, and multiple comorbidities, which significantly increased the risks of invasive procedures. Given that no pathogenic microorganism was identified in cultures, empiric broad-spectrum antibiotic therapy was initiated to address pulmonary infection. The focus of management was on supportive care for sepsis and bronchopneumonia. The patient was carefully monitored in the intensive care unit (ICU) for signs of worsening infection and hemodynamic instability with no hemorrhagic complications.

Despite these efforts, the patient developed multiorgan failure secondary to septic shock and succumbed to his condition five days later.

## Discussion

This case illustrates an AEF as a secondary complication of an aortic prosthesis implanted for aneurysm repair, a rare but recognized occurrence in 0.1% to 4% of cases [2]. The fistula was aorto-duodenal, a common location given the anatomical proximity of the aorta to the duodenum. In this case, the infected prosthesis is a key consideration as the potential origin of the fistula. Infection of the prosthesis, whether due to a postoperative hematoma infection, contamination, or delayed infection, can compromise the prosthetic integrity, weakening the anastomotic site and ultimately leading to the formation of an aortoenteric fistula.

Additionally, the presence of spondylodiscitis, a spinal infection, could have contributed to the development of the fistula. Spondylodiscitis often involves the vertebral bodies and can extend into surrounding structures, including the aorta. This can create a direct pathway for infection to spread, further compromising the aortic graft. In this patient, the combined effects of femoral prosthetic infection, potentially causing prosthetic contamination, and spondylodiscitis, which could have compromised the aorta, highlight the complex interplay of infections leading to the formation of the AEF [4].

In our case, the latency period was approximately 10 years, while the literature reports an average of 3 years, with extremes ranging from a few days to 15 years [1,2]. Although a delayed presentation of AEF is unusual, the complexity of the patient's medical history, coupled with the anatomical challenges, highlights the critical need for vigilance in the long-term follow-up of aortic graft patients.

AEF pathogenesis remains incompletely understood but is thought to involve chronic pulsatile stress, infection of a postoperative hematoma, or prosthetic contamination compromising the integrity of the anastomotic site. Side-to-end anastomosis has been associated with a higher risk than end-to-end configurations [5].

AEF diagnosis is particularly challenging due to its often subtle presentation. Gastrointestinal bleeding is the most frequent manifestation, commonly presenting as recurrent sentinel bleeding before massive hemorrhage. Other warning signs include acute abdominal pain, fever, anemia, leukocytosis, or a pulsatile abdominal mass, necessitating a high index of suspicion in patients with an aortic graft [5].

Radiologically, CT remains the modality of choice for AEF detection, offering sensitivity and specificity ranging from 40% to 90% and 33% to 100%, respectively [6,7]. CT angiography can reveal pathognomonic signs such as contrast extravasation, ectopic gas, periprosthetic collections, and loss of the fat plane. MRI provides comparable diagnostic accuracy but is less practical in emergencies. Ultrasound has limited value due to operator dependence and technical challenges. Angiography is primarily used for preoperative assessment or therapeutic embolization [8].

A thorough evaluation of clinical and imaging findings is required to differentiate AEF from perigraft infections, retroperitoneal fibrosis, infected aneurysms, and aortitis.

In AEF, there is a direct connection between the aorta (or graft) and the gastrointestinal tract, typically associated with ectopic gas and gastrointestinal bleeding. In contrast, perigraft infection is characterized by periaortic fluid collections, soft tissue stranding, and systemic inflammatory signs but does not involve direct enteric communication. Retroperitoneal fibrosis, a non-infectious fibro-inflammatory disorder, surrounds the aorta and ureters without fluid collections or systemic inflammatory markers. Although rare, infected aneurysms exhibit rapid expansion, irregular wall thickening, and systemic signs of infection, often originating from adjacent infectious sources such as spondylodiscitis. Inflammatory aortitis, on the other hand, presents with diffuse, concentric thickening and enhancement of the aortic wall, usually without aneurysmal dilation or nearby infection. The absence of ectopic gas and systemic inflammatory markers aids in distinguishing non-infectious conditions like retroperitoneal fibrosis [8,9].

Surgical intervention remains the cornerstone of AEF management, as the condition is almost universally fatal if left untreated [10]. While embolization can provide temporary stabilization in high-risk patients, it is rarely a definitive solution due to the risk of hemorrhage. Endovascular stenting can control bleeding but is associated with a high reinfection rate. In situ prosthetic reconstruction remains an option in cases of limited infection, but it carries significant recurrence and mortality risks (20%-56%) [11,12]. For patients with systemic sepsis, extra-anatomical bypass or arterial allografting may be necessary, although these techniques still have substantial early mortality rates of approximately 40% [9,13].

This case highlights the critical importance of long-term follow-up for patients with aortic grafts, especially those who may have a history of infection or other complicating factors such as femoral prosthetic infection or spondylodiscitis. The delayed presentation of the secondary aortoenteric fistula (AEF) in this case further emphasizes the need for vigilance in monitoring patients who may develop prosthetic infections, even years after initial surgery. The context of a chronic prosthetic infection, can significantly weaken the anastomosis and contribute to fistula formation, even after a long latency period. The management of AEF remains challenging, with high mortality rates if not addressed promptly.

## Conclusions

Radiology plays a critical role in the diagnosis and management of a secondary aortoenteric fistula (AEF), particularly in patients with a history of aortic graft placement and prior infections. This case underscores the necessity of vigilant long-term follow-up in individuals with comorbidities or previous infections, which may serve as entry points for pathogens, leading to prosthetic complications and AEF development. Given the patient's comorbidities, surgical intervention was not an option and a conservative management approach was adopted. Although this strategy was necessary due to the patient’s clinical state, it highlights the complexities of managing AEF in high-risk patients. Early detection, timely intervention, and a multidisciplinary approach remain crucial in managing this life-threatening condition, particularly when surgery is not feasible.
